# We Wrong Our Daughters

**Published:** 1877-08

**Authors:** 


### WE WRONG OUR DAUGHTERS.

We wrong them in that we compel them to marry. Our sons marry or not, as they please, whenever it suits their convenience, or whenever they can tease some body into taking them "for better or for worse," and the parents say it's all right; but they must marry off their daughters, get rid of them, and speedily, too, or they will be *old maids*, and so disgraced forever. The love of the parent succumbs to public opinion, to tyrant custom, and for fear of the "world's dread laugh," they send forth their young daughters into the soul-mart to be sold to the first, or more probably the highest bidder. Must not this be humiliating—galling—more bitter than rue?

The remedy for this wrong lies in giving your daughter some other aim in life except marriage, so that this may become to her a matter of will, not of necessity. Girls, as well as boys, ought to have something in view—something to stimulate them, something to bring out their energies. It is usual with parents to ask their sons, as soon as they are old enough to understand the question: "What do you intend to be?"

The boy's inclinations are watched, his tastes ascertained, his abilities weighed, in order that they may be better able to decide what shall be his future course. When his career is settled, all his powers are concentrated, all his energies directed to the accomplishment of that one object; his life becomes earnest, for he feels that he has a work to perform; he acquires a new dignity, for he is a person of importance in the world—he has a purpose in life; he is not a mere cipher. But what father among us, indulging and loving as he may be, turns from his proud boy, and while, perchance, a tear-drop glistens in his eye, lays his hand so tenderly on the broad white brow and silken tresses of his darling girl, and asks, with a strange tremor in his manly voice: "And what is my heart's child going to be?" If ever such a thought crosses his mind, it usually amounts to nothing more than: "She will be a belle, and make a great match." Thus, in every instance, the one everlasting and apparently inevitable idea of marriage, as though no woman had ever lived and died without being married, or without even desiring to be. I can not see why girls should be brought up to the idea that marriage is the "one thing needful," the "*summum bonum*," the "nothing more beyond." I wish they would begin to think otherwise.—

